# Types of kindergarten and their relationship with parental and children’s socio-demographic characteristics in Denmark

**DOI:** 10.1371/journal.pone.0288846

**Published:** 2023-07-20

**Authors:** Ina Olmer Specht, Sofus Christian Larsen, Ann-Kristine Nielsen, Jeanett Friis Rohde, Berit Lilienthal Heitmann, Tanja Schjødt Jørgensen

**Affiliations:** 1 The Parker Institute, Bispebjerg and Frederiksberg Hospital, Frederiksberg, Denmark; 2 Unit for General Practice and Section of General Practice, Department of Public Health, University of Copenhagen, Copenhagen, Denmark; 3 The Boden Group, The Charles Perkins Centre, Faculty of Medicine and Health, University of Sydney, Camperdown, Australia; Rutgers The State University of New Jersey, UNITED STATES

## Abstract

In Danish outdoor kindergartens, children are spending most of the day outdoors often in forests or similar nature environments. These children are assumed to be healthier than children attending conventional kindergartens, however, factors related to choosing a specific type of kindergarten may explain the differences. To better understand this, we aimed to investigate parents reasons for choosing either outdoor or conventional kindergartens based on a mixed-method participatory Concept Mapping approach, and further if parental socio-demographics and early child characteristics differed prior to enrolling children to either type of kindergarten using a cohort register-based approach.

Parents of children attending outdoor kindergartens (n = 23) weighed reasons such as “physical setting, outdoor life, and freedom of movement” high, whereas “a good first impression of the kindergarten” was an important reason for parents choosing a conventional kindergarten (n = 22). In the register-based approach, 2434 and 2643 children attended outdoor or conventional kindergartens, respectively. The parents choosing outdoor kindergartens as well as their children differed according to most investigated characteristics, including origin (maternal non-Western: 4.2% vs. 21.9%, p < .0001), educational level (maternal long education: 45.6% vs. 33.0%, p < .0001), prematurity (5.1% vs. 7.1%, p = 0.004) and sex (females: 43.5% vs. 48.6%, p = <0.0013). In conclusion, parental reasons for choosing kindergarten as well as parental socio-demographics differed substantially among kindergarten type. These differences might cause selection bias if not considering when comparing health outcomes among children attending different kinds of kindergartens.

## 1 Introduction

In the Scandinavian countries, up to 97% of children at the age of three to five years are attending kindergartens [1], whereas the average enrolment rate in the Organization for Economic Cooperation and Development (OECD) counties is 87% [2]. Since many pre-school children attend up to 40 hours of kindergarten per week [3] kindergartens are institutions that contribute substantially to the secondary socialisation of the child as well as to the daily amount of physical activity. Kindergartens can thereby be considered ideal settings for a structural way to improve public health initiatives in young children.

In the Scandinavian countries there are overall two types of kindergartens with the same ratio of personnel per child: the conventional kindergarten and the outdoor kindergarten. In the conventional kindergarten children are spending time both indoor playing with toys, drawing and tumbling, and with options for outdoor activities in the kindergarten playground. Whereas in the outdoor kindergarten, a Danish invention from 1952, almost all hours during the day are spent outdoor, usually in larger forests or in rural areas without a formal playground [4–6]. Children attending outdoor kindergartens are often living in the bigger cities and together with the kindergarten group they are transported by bus from the city to the outdoor kindergarten. This type of kindergarten is most often inflexible in relation to when the parents can bring and pick up the child at the collection point in the city. Outdoor kindergartens are generally a Scandinavian institution form but are getting increasingly popular in other countries like Holland, Germany, Canada, and Australia. Outdoor kindergartens go under many names among others; nature kindergartens, forest kindergartens, bush kindergartens or Waldkindergartens, however, the philosophy remains the same namely spending as much time as possible outdoor in the nature.

In Denmark, it is a public opinion that children from outdoor kindergartens often have better motor development, are more physically active and less sick [7]. The evidence for these health benefits is, however, limited although favour a benefit [8–13]. A Norwegian quasi-experimental study investigated motor ability over a period of 9 months [9]. The study included 46 children in the experimental group from one kindergarten and offered them 1–2 hours of free play in a forest, and a reference group of 29 children from two other kindergartens who were offered 1–2 hours of free play in the kindergarten playground [9]. Balance and coordinating abilities were found to be significantly increased in the experimental group compared with the reference group. Another study from Sweden, including 197 children from 11 kindergartens, showed that the children attending kindergartens with a large outdoor play area with many trees, had a higher step count than children attending kindergartens with smaller outdoor environments [11]. However, results from some of the previous studies might have been biased due to selection, since reasons for parents seeking the outdoor kindergarten, parental demographic, or child health prior to starting kindergarten were often not controlled for [14, 15].

If outdoor kindergartens in fact are health promoting, it would seem an easy way to improve health among young children. It is therefore of importance to investigate what factors determine choice of kindergarten type, to be able to optimize the use of outdoor kindergartens among parents. The overall aim of the present study was thus to investigate parental reasons for choosing an outdoor or a conventional kindergarten, and to investigate if the parents differed according to socio-demographic factors. This was done using two study design; first via a mixed-method participatory Group Concept Mapping (GCM) approach, to investigate parental reasons/considerations for choosing either an outdoor or a conventional kindergarten, and second, using a register-based approach, to examine socio-demographic characteristics among parents and early child health and characteristics prior to kindergarten admission.

## 2 Materials and methods

The present study was part of the ‘Outdoor kindergartens—the healthier choice?’ (ODIN) study which was initiated with the main objectives to investigate pedagogic and didactic practise as well as health effects including physical activity, sleep and infections requiring antibiotics and long-term outcomes like weight, growth and motor development, among children attending outdoor kindergartens compared to children attending conventional kindergartens.

Outdoor and conventional kindergartens differ by their environmental setting by a larger outdoor area often in forest settings for the outdoor kindergarten. The children and kindergarten teachers spend most of the day outside in the outdoor kindergarten and nature and motor development are often pedagogic areas of interest [6].

In the following sections, we describe the two methods used to investigate (1) parental reasons for choosing type of kindergarten (a General Concept Mapping design) and (2) the socio-demographic characteristics of parents and children at kindergarten enrolment (a register-based design).

We followed the Strengthening the Reporting of Observational Studies in Epidemiology (STROBE) guidelines for reporting observational studies.

### 2.1 The Group Concept Mapping (GCM) study

GCM is a formal group process using a structured approach to identify ideas on a topic of interest and organize them into domains based on a mixed-method participatory design that incorporates group processes and multivariate statistical analyses (multidimensional scaling and hierarchical cluster analysis) [16, 17]. GCM is considered highly effective for developing outcome measures (e.g. patient perceptions when investigating health care) [18–20]. Within GCM, participants are involved in several steps of the conceptual process and the final results are illustrated in maps where ideas developed during the process are organized thematically [21]. The GCM process includes the following phases: 1) preparation and generation of seeding question by the researchers, 2) generation of statements (individual brainstorming done by the participants), 3) structuring statements (sorting and rating of importance of statements done by the participants), 4) program preforming GCM analysis (sorting results from step 3 serves as an input to the multidimensional scaling and to the creation of maps [19], 5) interpreting the result (validation done by the participants at a face-to-face meeting facilitated by the researchers), and 6) utilization of the result.

#### 2.1.1 Study participants and settings in the GCM

Parents to children attending either outdoor or conventional kindergartens were invited to participate in two cluster online GCM workshops using the Concept System® Groupwisdom^TM^ software, designed to support each step in the GCM process, and in one face-to-face group session for each of the two groups at the Parker Institute, Bispebjerg and Frederiksberg Hospital, Denmark, in the period January 2021 to June 2021. Parents were consecutively enrolled, some before study start and some during later stages of the GCM. Parents were recruited through their children’s kindergarten or through social media, thus the number of parents seeing the invitation to participate was unknown.

Which online and physical workshop the parents attended depended on the type of kindergarten their child attended. The parents in the two kindergarten groups were not aware that they were selected into either the outdoor or the conventional kindergarten groups and were all asked the same initial open-ended question: ‘*Thinking as broadly as you can*, *please list your thoughts regarding which considerations you had when choosing a kindergarten for your child*?’. Clustering analysis was performed based on the participant statements based on the initial question using multidimensional scaling analysis (CS Global MAX; Concept Systems, Inc. [22]). Any duplicate statements were removed [19–21, 23].

To identify which statements based on the open-ended question were of most importance for the parents when choosing kindergarten, parents were asked to rate the importance of each statement on a 5-point scale, from 1 (‘not important’ for choosing kindergarten) to 5 (‘very important’). Mean and median ratings of importance assigned by the parents for each statement were calculated using Wilcoxon two-sample test.

Based on the sorting and rating, multidimensional scaling and cluster analyses were performed, in which related statements were grouped into concepts [24]. Within the multidimensional scaling analysis, a ‘stress value’ was used to indicate congruence between the raw data and the processed data (goodness of fit statistic) (13). A low stress value (<0.39) is considered readily interpretable. During the cluster analyses, several cluster solutions were generated, and the one that matched the data the best (i.e., the cluster solution representing sufficient details on the topic) was applied, creating the Cluster Map.

At the face-to-face validation session facilitated by the researchers, parents met to interpret and validate the results created in the earlier online stages of the GCM process. Based on the Cluster Map and an overview of concepts and statements generated online by the parents, the parents were instructed to (a) determine if each statement was placed in the right cluster, (b) consider the number of concepts, and (c) label each concept so that each cluster illustrated the theme of the cluster. Reflections and suggestions were discussed to obtain consensus.

After the validation session, all parents attending the GCM stages were contacted either by phone or e-mail and asked to answer a list of questions related to their level of education and living area of residence, as well as the gender of the child and parent, and the number of siblings.

### 2.2 The register-based study

The study population from the GCM was not part of the study population in the register-based study.

#### 2.2.1 Study population in the register-based study

The exploratory register-based study was based on a cohort of n = 5077 children attending either outdoor or conventional kindergartens, obtained from the Copenhagen Municipality and Aarhus Municipality, the two largest cities of Denmark. Both municipalities provided personal identification numbers (CPR-numbers) of the children and health information gathered by health nurses during the first year of the child’s life and from the school health surveys from children who went to an outdoor kindergarten in the period 2011–2019 and from a random subsample among all children from conventional kindergartens living in the same areas of parental residence as the outdoor kindergarten children.

To be eligible, the participants had to have attended either an outdoor kindergarten or a conventional kindergarten in the years they lived in the municipality, thus the children moving in or out of the municipality had a shorter duration of kindergarten exposure. This data was merged with data on demographics and health of parents and children gathered from the Danish Medical Birth Registry, the National Patient Registry and Statistics Denmark. In this present study, all information prior start in kindergarten was included.

#### 2.2.2 Outcome assessment in the register-based study

CPR and health nurse data from the municipalities were sent to Statistics Denmark where merging at the individual level with information from the Danish Medical Birth Registry, the National Patient Registry and Statistics Denmark took place. Data from the Danish Medical Birth Registry provided information on maternal pre-pregnancy Body Mass Index (BMI, kg/m^2^), parental age at birth (years), smoking during pregnancy (yes/no), complications during pregnancy (yes/no), multiple pregnancies (yes/no), caesarean section (yes/no), sex of the child (female/male), gestational age (days), and birth weight (grams). Data on child hospital admissions (nr. of admissions) were collected from the National Patient Registry. Information on emergency room admissions (nr. of admissions) were only available until 31.12.2013 thus data on children starting kindergarten after 2013 were lacking. Data on parental education (Basic [basic school 8th–10th class]; Short [general upper–secondary education, short-cycle higher education or vocational education and training]; Medium [medium-cycle higher education or bachelor]; and Long [long-cycle education and PhD]), and origin (Western/non-Western) were gathered from Statistics Denmark. Western/non-Western countries are defined by Statistics Denmark. Western counties are countries within the EU and associated countries as well as the four Anglo-Saxon counties.

#### 2.2.3 Statistical analysis in the register-based study

Characteristics of children and parents from outdoor and conventional kindergartens are presented descriptive as number (n) and percentage (%) for categorical variables and mean and standard deviations (SD) for continuous variables. The two groups were compared using the chi-square (χ2) test or the t-test, depending on whether the outcome variable was discrete or continuous, respectively.

In an ad hoc analysis we further investigated whether education was associated with the other investigated co-variates.

All analyses were performed in SAS Enterprise Guide 8.3 on a secure platform at Statistics Denmark, and all statistical tests were two-sided with a significance level at 0.05.

### 2.3 Ethical considerations

Permission to send individual data on CPR and health nurse information to Statistics Denmark was granted by the two municipalities.

Permission from the Ethical Committee was evaluated not to be relevant (journal nr.: H-19053587). Permissions from the Capital Region Data Agency and the Danish Patient Safety Authority were granted (Journal nr.: P-2020-54 and 31-1521-8, respectively). Parents in the GCM study gave written consent before study start and all parents were informed about their right to withdraw from the study at any time. Registries relevant for the register-based study were accessible through a secure platform at Statistics Denmark, and the collection of data from these registries was in accordance with accepted ethical principles for informed consent according to the Declaration of Helsinki.

## 3 Results

### 3.1 The GCM study

In the GCM study, overall, 45 parents agreed to participate, 23 (51%) in the outdoor kindergarten group and 22 (49%) in the conventional kindergarten group.

Of the 23 parents in the outdoor kindergarten group, four did not register for the online GCM program. Five out of the 23 parents were snowballed after the online GCM process had started, thus only attending the sorting and rating stages and the face-to- face validation meeting. Sixteen parents finished the brainstorm stage, 14 the sorting stage, 13 the rating stage and seven the face-to-face meeting.

Of the 22 parents in the conventional kindergarten group, three parents were snowballed after the online process had started, thus only attending the sorting and rating stages and the face-to-face validation meeting. Eighteen parents finished the brainstorm stage, 15 the sorting stage, 16 the rating stage and six the face-to-face meeting.

Among the 23 and the 22 parents in the outdoor and the conventional kindergarten groups, respectively, 18 responded in both groups to the demographic questions (78% and 82%, respectively, Table 1). Among those 18 parents in both groups, 13 (72.2%) were women, and the majorities had a long education (n = 13 [72.2%] and n = 11 [61.1%], respectively). In total, 14 (77.8%) of the children from the outdoor kindergarten and 17 (94.4%) from the conventional kindergarten had siblings.

**Table 1:** Demographic information of parents from the outdoor kindergarten and the conventional kindergarten groups.

	Outdoor kindergartens n = 18		Conventional kindergarten n = 18	
	n	%	n	%
Female gender of the parent	13	72.2	13	72.2
Education				
Short	1	5.6	1	5.6
Medium	4	22.2	6	33.3
Long	13	72.2	11	61.1
Having siblings	14	77.8	17	94.4
Living in the Copenhagen area	18	100	14	77.8
Female gender of the child	6	33.3	9	50.0

A total of 97 and 132 statements were generated by the parents in the outdoor kindergarten group and the conventional kindergarten group, respectively. After removing redundant statements (i.e., ideas with the same wording or meaning) and minor linguistic revisions (i.e., dividing sentences with several statements in one sentence), 84 and 87 unique statements remained for the sorting and rating stages, respectively.

The multidimensional scaling analysis revealed low stress values of 0.19 and 0.21, respectively, indicating that the cluster rating maps were readily interpretable, and we therefore proceeded with generating the cluster rating maps. After carefully reviewing the various cluster rating maps, we identified the 8-cluster solution as the optimal solution for the outdoor kindergarten and the 9-cluster solution as the optimal solution for the conventional kindergarten (S1 and S2 Figs).

At the face-to-face validation meeting, discussions led to a consensus about the location of the statements, the number of concepts and the labels for each concept. In the revised maps the outdoor kindergarten and the conventional kindergarten groups generated 6 and 8 concepts, respectively. Each concept contained between six and 27 statements related to the outdoor kindergarten and 6 and 19 statements related to the conventional kindergarten (S1 and S2 Tables).

The six concepts generated by the outdoor kindergarten group; ‘physical setting with a focus on outdoor life and freedom of movement’, ‘learning/freedom and pedagogical ideology’, ‘personnel’, ‘everyday life’, ‘experiences’, and ‘structure and organization’, are summarised in Table 2. The parents in the outdoor kindergarten group ranked the concept “Physical setting with a focus on outdoor life and freedom of movement” with a mean score of 3.9 out of a maximum of 5 as containing the most important statements. Whereas the concept with less important statements was “Experiences” with a mean score of 2.8 (Table 2).

**Table 2:** Description of the six concepts from the outdoor kindergarten group.

Concept	No. of statements (%)	Concept mean / median (min-max)	Summary of content
1. Physical setting with a focus on outdoor life and freedom of movement	27 (32.1%)	3.9 / 4 (3–5)	More time spent outside. The large outdoor area, nature, and more room for play, also without constant adult supervision. The health of being outside in the nature and fresh air, especially when living in the city. Also, the importance of a nice indoor area in the kindergarten.
2. Learning, freedom, and pedagogical ideology	19 (22.6%)	3.4 / 4 (2–5)	Focus on inclusive, playing, smiling and active kindergarten teachers, who helps the children develop. The importance of a good communication between parent and the kindergarten teachers and the managers of the kindergarten.
3. Personnel	12 (14.3%)	3.8 / 4 (3–5)	A good first impression of the employees in the day care. Long seniority of kindergarten teachers and the managers.
4. Everyday life	11 (13.1%)	3.2 / 3 (2–4)	The distance from home to the pickup point of the bus should be short due to the logistic of the everyday life. Also focus on a not too long transportation time from the pickup point to the outdoor kindergarten.
5. Experiences	9 (10.7%)	2.8 / 3 (1–4)	Good recommendations from friends and acquaintances whose children have attended the institution. An institution with not too many children, and with children from the area of their own residence.
6. Structure and organization	6 (7.1%)	2.9 / 3 (3)	The importance of the organizational structure with a flat management, focus on safety, and well organized.

The parents from the outdoor kindergartens ranked *“Kindergarten teachers and their pedagogic approach to children”*, “*Playing*, *smiling*, *and attentive adults*”, "*Present kindergarten teachers*" and “*Kindergarten teachers with a focus on well-being*”, as being the most important statements (Table 3). In contrast, statements such as, “*Leader with long seniority*”, “*Wanted a small kindergarten in relation to the number of children*", “*Divided zones for play activities*", and “*I attended an outdoor kindergarten myself as a child*.” were ranked as being of minor or no importance (Table 3).

**Table 3:** Examples of ranking of statements from the outdoor kindergarten group. All statements are presented in S1 Table.

Concept no.	Ranking of statements	Importance*
2	Kindergarten teachers and their pedagogic approach to children.	4.6
2	Playing, smiling, and attentive adults.	4.6
3	Present kindergarten teachers.	4.6
2	Kindergarten teachers with a focus on well-being.	4.6
1	Activity, space to run, climb trees, dig, jump, etc.	4.3
3	Stable staff with long seniority.	3.9
5	Other parents’ recommendations.	3.2
3	Leader with long seniority.	2.6
5	Wanted a small kindergarten in relation to the number of children.	2.6
2	Focus on posters and other information relevant for the children.	2.1
2	Divided zones for play activities.	1.9
5	I attended an outdoor kindergarten myself as a child.	1.5

*5: very important; 4: important; 3: moderate importance; 2: minor importance; 1: not important

The eight concepts generated by the conventional kindergarten group; ‘Physical framework’, ‘Structure / pedagogical values / food policy’, ‘Staff and workplace’, ‘Atmosphere’, ‘Reputation’, ‘Logistics’, ‘Shifts’ and ‘Staff-to-child ratio’, are summarised in Table 4. The parents in the conventional kindergarten group ranked the concept “Atmosphere” with a mean score of 3.8 as the most important concept. Whereas the concept with less important statements was “Staff-to-child ratios” with a mean score of 2.8 (Table 4).

**Table 4:** Description of the eight concepts from the conventional kindergarten group.

Concept	No. of statements (%)	Concept mean / median (min-max)	Summary of content
1. Physical framework	19 (16.5%)	3.1 / 3 (1–4)	Good indoor and outdoor facilities. Indoor activity rooms to tumble in.
2. Structure / pedagogical values / food policy	17 (14.8%)	3.3 / 4 (3–5)	A good arrangement of roles among the kindergarten teachers in the groups. Clear pedagogical and social values among the personnel. Room for creativity. Focus on organic homemade food.
3. Staff and workplace	13 (11.3%)	3.7 / 4 (2–5)	A good communication among staff, parents, and children. Employees with long seniority.
4. Atmosphere	9 (7.8%)	3.8 / 4 (2–5)	If the first impression of the kindergarten was good with a good atmosphere.
5. Reputation	9 (7.8%)	2.9 / 3 (2–3)	The importance of good recommendations from other parents and friends.
6. Logistics	8 (7.0%)	3.7 / 4 (2–5)	The flexibility in relation to drop-off and pick-up, thus outdoor kindergartens were not on options due to strict drop-off and pick-up times. An institution close by the residence.
7. Shifts	6 (5.2%)	3.6 / 4 (2–5)	The importance of choosing an integrated institution with both nursery and kindergarten for easy transition between the two. A kindergarten in the same district as the primary school.
8. Staff-to-child ratios	6 (5.2%)	2.8 / 4 (2–4)	A small kindergarten with a low number of children per employee.

The parents from the conventional kindergartens ranked the statements “*Present and committed staff*”, “*The general atmosphere of the institution is important*”, "*That the institution was located close to where we live*”, “*That the kindergarten teachers seemed happy and spoke warmly about the kindergarten*, *the personnel and the management*”, as being the most important statements (Table 5). In contrast, statements such as, “*Small cozy institution with few groups of children*”, “*We looked at the parental satisfaction survey*", “*That the kindergarten was in close proximity to the city oasis*, *playgrounds*, *parks*, *etc*.", and “*That the kindergarten had an outdoor kitchen so they could cook for the kids on the playground*” were ranked as being of minor or no importance (Table 5).

**Table 5:** Examples og ranking of statements from the conventional kindergarten group. All statements are presented in S2 Table.

Concept no.	Ranking of statements	Importance*
3	Present and committed staff.	4.7
4	The general atmosphere of the institution is important.	4.5
6	That the institution was located close to where we live.	4.4
3	That the kindergarten teachers seemed happy and spoke warmly about the kindergarten, the personnel, and the management.	4.4
8	The number of children compared to the number of adults to be with the children.	3.6
1	The indoor facilities.	3.5
3	That the employees had been employed for a long time.	3.0
2	Organic food, made in the kitchen of the institution.	3.1
3	That there were male kindergarten teachers.	2.6
1	Small cozy institution with few groups of children.	2.4
5	We looked at the parental satisfaction survey.	2.1
1	That the kindergarten was in close proximity to the city oasis, playgrounds, parks, etc.	2.1
1	That the kindergarten had an outdoor kitchen so they could cook for the kids on the playground.	1.5

*5: very important; 4: important; 3: moderate importance; 2: minor importance; 1: not important

### 3.2 The register-based study

In the exploratory register-based cohort study, 2434 children had attended outdoor kindergartens and 2643 children had attended conventional kindergartens. As shown in Table 6 parents characteristics differed for the majority of the investigated socio-demographic characteristics; both maternal and paternal education were higher among children attending outdoor kindergartens (p<0.0001) and more parents with non-Western origin chose outdoor kindergartens (p<0.0001). Also, most early child characteristics differed according to type of kindergarten, with fewer children born preterm, and fewer delivered by caesarean section among children from outdoor kindergartens (5.1% vs. 7.1%, p = 0.031 and 18.7% vs. 21.1%, p = 0.04, respectively). Also, fewer girls than boys attended outdoor kindergartens (43.5% girls vs. 48.6% p = <0.001, Table 6), but we found no statistically significant differences related to kindergarten type in none-specified hospital admissions (p = 0.099), or emergency department admissions (p = 0.755), however, the latter only included children starting kindergarten prior to 2014 (n = 142).

**Table 6:** Demographic and health characteristics among children and parents before starting either outdoor or conventional kindergartens.

	Outdoor kindergartens, n = 2434				Conventional kindergartens, n = 2643				t-value or χ2^1^	p-value
	**n**	**%**	**Mean**	**SD**	**n**	**%**	**Mean**	**SD**		
*Residence*									5.64	0.018
Copenhagen Municipality	1473	60.5			1685	63.6				
Aarhus Municipality	961	39.5			958	36.3				
*Parental characteristics*										
Maternal origin, non-Western	102	4.2			572	21.9			337.63	<0.0001
Paternal origin, non-Western	120	5.1			538	21.2			271.40	<0.0001
Maternal education^2^									175.81	<0.0001
Basic	118	5.2			324	13.7				
Short	410	18.1			619	26.2				
Medium	698	30.8			638	27.0				
Long	1038	45.6			780	33.0				
Paternal education^2^									99.33	<0.0001
Basic	160	7.3			329	14.4				
Short	648	29.6			808	35.3				
Medium	488	22.3			450	19.7				
Long	896	40.9			701	30.6				
Maternal smoking during pregnancy	151	6.8			223	9.5			11.28	<0.001
Maternal pre-pregnancy BMI (kg/m^2^)	2236		22.4	3.4	2355		23.2	4.2	7.17	<0.0001
Maternal age at pregnancy (years)	2335		31.8	4.7	2453		31.1	4.9	-5.07	<0.0001
Paternal age at pregnancy (years)	2278		34.0	5.7	2399		33.8	6.3	-1.18	0.238
Parity									7.07	0.008
Parity 0	1324	58.6			1310	54.7				
Parity ≥1	935	41.4			1083	45.3				
Complications during pregnancy	248	4.5			273	4.9			0.11	0.737
*Child characteristics*										
Multiple pregnancies	74	3.0			100	3.8			2.12	0.145
Caesarean section	455	18.7			558	21.1			4.64	0.031
Sex of the child									13.31	<0.001
Female	1059	43.5			1285	48.6				
Male	1375	56.5			1358	51.4				
Preterm birth	115	5.1			170	7.1			8.19	0.004
Gestational age at birth (days)	2276		279.6	11.8	2411		277.8	12.7	-4.89	<0.0001
Birth weight (grams)	2264		3497	527	2400		3442	550	-3.42	<0.001
Age at kindergarten registration (years)	2325		3.3	3.7	2513		3.0	3.4	1.90	0.046
Hospital admissions (n)	1439		4.6	4.3	1563		4.9	4.2	1.65	0.099
Emergency room admission^3^ (n)	77		1.5	0.8	65		1.5	0.8	0.31	0.755

^1^ t-values are presented in continues variables and χ2 in discrete variables.

^2^ Education: Basic [basic school 8th–10th class]; Short [general upper–secondary education, short-cycle higher education or vocational education and training]; Medium [medium-cycle higher education or bachelor]; and Long [long-cycle education and PhD].

^3^only includes children starting kindergarten in the period 2011–2013 since data only was available from this period.

## 4 Discussion

The objectives of this study were to identify (1) parental reasons for choosing either an outdoor or conventional kindergarten, and (2) differences in parental socio-demographic and early child characteristics before attending an outdoor or a conventional kindergarten. To investigate this, two different methodological approaches were used not including the same study participant; a GCM approach inviting parents with children attending an outdoor kindergarten or a conventional kindergarten, and a register-based cohort.

The GCM approach showed that parents of children attending outdoor kindergartens weighed values related to outdoor and physical settings high, whereas a good first impression of the kindergarten was the most important reason for choosing the conventional kindergartens. Several of the concepts contained the same types of reasons for choosing kindergarten among the two groups, like the concepts ‘Everyday life’ and ‘Logistics’ which related to it being important that kindergartens were close to home, but also with a clear deselecting of the outdoor kindergartens by the parents from conventional kindergartens due to difficult logistics of bringing and picking up children at specific time points. However, the differences among the two groups were also apparent especially in relation to the environmental settings, with a higher focus on the outdoor environment, physical activity and nature among parents to children attending outdoor kindergartens, while the focus was more related to the indoor opportunities for tumbling and creativity for parents to children from the conventional kindergarten group. Also, only parents with children attending conventional kindergartens had a focus on the staff-to-child ratios, although this was the cluster with the lowest mean score. The number of children per kindergarten teacher or personnel (staff-to-child ratio) has decreased in Denmark the last years due to a lack of educated kindergarten teachers and thus been highly debated since it might influence the everyday life of the child and employee [25]. The Danish media have thus had a high focus on this ratio, and parental organisations have been developed to pressure the government and the municipalities to decrease the number of children per kindergarten staff. Interestingly, the results from this study pointed toward other values among the parents for choosing kindergartens, like the size of the kindergartens, attentive, welcoming, and caring kindergarten teachers, and personnel and management with long seniorities. Conflicting the parental reasons for choosing type of kindergarten, many Danish municipalities are currently merging smaller kindergartens into larger kindergartens to save costs. Also, many Danish kindergartens are having troubles keeping and employing kindergarten teachers due to lack of educated personnel [26]. This has resulted in more employees currently without a pedagogic training [27], constituting often younger people working for a shorter period (e.g. before starting an education), and a large flow of new personnel.

The register-based approach showed that the parents choosing outdoor kindergartens more often had a Western origin, a lower pre-pregnancy BMI, smoked less during pregnancy, and gave birth at term. However, in an ad hoc analysis we found that all variables except preterm birth were related to the higher educational level among parents choosing outdoor kindergartens, which also has been shown by others [28].

Previous studies have shown that children from less affluent homes tend to be less physically active in their spare time [29], spend less time outdoors, and use nature less than children from more affluent families [30]. Since outdoor play is a major source of physical activity among children [31, 32], children from less affluent families can be expected to benefit most from outdoor kindergartens, however according to our results in the register-based cohort study and previous studies [33, 34], few such children attend outdoor kindergartens. Overall, a decline in outdoor play over time has been shown [35] despite evidence for health benefits from outdoor play [36–38]. However, whether child attendance at outdoor kindergartens promote health is still unknown, since most of the previous studies investigating nature contact and children’s health seems biased due to selection or confounding due to lack of comparability among investigated groups [14]. We did, however, show in a newly published study that children attending rotating kindergartens (kindergartens where the children biweekly were in their outdoor kindergarten or their conventional kindergarten) took more steps when in the outdoor kindergarten [39]. If attending outdoor kindergartens in fact is health promoting as suggested by some [8–13] and results are not biased, outdoor kindergartens could be a way of more outdoor play, and then a strategy on how to optimise the use of outdoor kindergartens, especially among less affluent families, would be highly relevant. Based on our results from the GCM study, the major reason for not choosing an outdoor kindergarten was logistics in relation to the bus ride to and from the outdoor kindergarten area. To increase the use of outdoor kindergartens, finding new or more flexible solution to transporting the children may be needed. However, the parents from our GCM study and the parents from the register-based study might not be comparable. The parents from the register-based study were not self-selected into the study like the GCM parents but were randomly selected by the municipalities that provided the data. The GCM parents had a higher educational level and might also be more interested in the early child educational system.

More boys than girls attended outdoor kindergartens in the register-based study. Indeed, parents might to a higher degree choose an outdoor kindergarten if they have an active son. In accordance, a previous study showed that boys generally seem to be more active during day care hours than girls [40]. Also a study investigating 476 children aged seven to nine years showed that parents allowed boys to play outdoors alone in a younger age than girls [41], and parents seem to be more protective of their toddler daughters than sons [42]. In our GCM study, parents choosing outdoor kindergartens often valued physical activity more than parent choosing conventional kindergartens, as indicated by statements like ‘Be allowed to be an active child’ and ‘Activity, space to run, climb trees, dig, jump, etc.’, whereas parents choosing conventional kindergartens did not focus on physical activity per se but more on opportunities for creativity, and gave statements like ‘That the kindergarten emphasize creative activities’ and ‘That there was a special focus (on creativity and art) was positive’. The observed gender difference could also be social-culturally determined by parent’s believes about gender roles, expecting boys to have higher needs for activity and girls higher needs for creativity [43], leading to differences in physical activity and outdoor play [40].

The present study has several advantages including the large sample size in both the GCM study and the register-based study. In the register-based study we included all children who had attended an outdoor kindergarten in the period 2011–2019 and were living in the Copenhagen or Aarhus municipalities, the two largest cities of Denmark, and selected a sub-sample randomly among children attending conventional kindergartens from the same area of residence. In the GCM approach a large number of statements were generated, and the fact that a relatively large number of statements were redundant indicated that the number of statements was sufficient to reach data saturation. The redundancy was also illustrated in the calculated stress value, which was comfortably below the commonly accepted threshold. The GCM includes the voice and involvement of the participants; the data are thus not research generated but involved the participants in all phases from generation of data, through data analysis, and to validation of results. However, both approaches have some potential limitations including a potential low generalizability. The majority of the parents in the GCM approach had residence in the Capital Region of Denmark, and also in the register-based study we only had information from two Danish municipalities which might not represent parents and children from other cities or countries. Also, the parents included in the GCM might not be comparable with the parents in the register-based study. Parents in the GCM were committed and better educated than parents in the register-based study, which is a major challenge in many studies with active participation [44]. However, although the results might not be generalisable to other countries, this study did show differences in parental values when selecting kindergartens as well as major differences in socio-demographics. This might also be relevant information for other counties and is important to consider when investigating health benefits of different types of kindergartens.

## 5 Conclusion

Our results suggest that parental choice of kindergarten is related to considerable differences in both reasons for choosing type of kindergarten, as well as socio-demographics, and early child characteristics. Outdoor life and nature ranked higher among parents choosing an outdoor kindergarten without focusing on the strict dropping off and picking up times, while a clear deselection of the outdoor kindergartens was apparent for that particular reason among parents choosing conventional kindergartens. These parents rather had a high focus on the initial atmosphere when visiting the kindergarten for the first time, the pedagogic values, and the room for indoor activities. In the register-based study, the parents and children attending the two types of kindergartens differed according to most of the investigated socio-demographic and early child characteristics. These differences might have caused major selection bias when previous studies compared beneficial health outcomes for children attending different kinds of kindergartens and are thus important to consider in future studies.

## Supporting information

S1 Table Conceptual model. All statements were generated, ranked and divided into concepts by the parents from children attending conventional kindergartens.
(PDF)

S2 Table Conceptual model. All statements were generated, ranked and divided into concepts by the parents from children attending outdoor kindergartens.
(PDF)

S1 Fig The optimal cluster solution for statements generated among parents with children attending outdoor kindergartens. (PDF)

S2 Fig The optimal cluster solution for statements generated among parents with children attending conventional kindergartens. (PDF)
