# Supplementary material for: Types of kindergarten and their relationship with parental and children’s socio-demographic characteristics in Denmark
Source: PLoS One. 2023 Jul 20;18(7):e0288846. doi: 10.1371/journal.pone.0288846 (PMC10358886; doi:10.1371/journal.pone.0288846)
Supplement: S1 Table — All statements were generated, ranked and divided into concepts by the parents from children attending conventional kindergartens. (PDF) [file pone.0288846.s001.pdf]

**S1 Table.** Conceptual model. All statements were generated, ranked and divided into concepts by the parents from children attending conventional kindergartens.

| Thinking as broadly as you can, please list your thoughts regarding which considerations you had when choosing a kindergarten for your child? | **The Frequency of ranking*** | | | | | **Statement** | | **Cluster** | |
|---|---|---|---|---|---|---|---|---|---|
| **Statements** | **1** | **2** | **3** | **4** | **5** | **Mean** | **Median** | **Mean** | **Median** |
| **ATMOSPHERE** | | | | | | | | | |
| The general mood of the institution is important | | | | 7 | 8 | 4.5 | 5 | 3.9 | 4 |
| Does the personnel from other parts of the kindergarten greets and does the kindergarten manager welcome you. | 1 | 3 | 4 | 6 | 1 | 3.2 | 3 | | |
| That there was a good atmosphere when we were to visit the institution | | | | 9 | 6 | 4.4 | 4 | | |
| How the kindergarten teachers we met on the tour in the kindergarten spoke to the kids | | 1 | 1 | 6 | 7 | 4.3 | 4 | | |
| We would like to choose an institution where the children reflected our very mixed local area, so that our child meets children who have a different background than herself. | 5 | 3 | 4 | 2 | 1 | 2.4 | 2 | | |
| We emphasized a calm atmosphere among children and adults | | 2 | 4 | 5 | 4 | 3.7 | 4 | | |
| Visited the institution before my kids had to start so I could get an insight into what weekdays in the institution were like. | 1 | | 2 | 10 | 2 | 3.8 | 4 | | |
| That we got a good impression of the kindergarten after the first visit of the kindergarten | | | 2 | 7 | 6 | 4.3 | 4 | | |
| That there was a good atmosphere and mental surplus among the staff | | | 1 | 8 | 6 | 4.3 | 4 | | |
| **STAFF AND WORKPLACE** | | | | | | | | | |
| Present and committed staff | | | | 5 | 10 | 4.7 | 5 | 3.6 | 4 |
| That the management was respected by the employees | 2 | | 6 | 6 | 1 | 3.3 | 3 | | |
| That the employees had been employed for a long time | 2 | 2 | 6 | 4 | 1 | 3.0 | 3 | | |
| That there were male kindergarten teachers | 2 | 6 | 4 | 2 | 1 | 2.6 | 2 | | |
| How large a proportion of the staff were pedagogically trained | 3 | 2 | 1 | 6 | 3 | 3.3 | 4 | | |
| It made an impression on us that the leader of the chosen institution knew all the children we met during the tour | 2 | 5 | 4 | 3 | 1 | 2.7 | 3 | | |
| We emphasized that the leader seemed sympathetic and committed to the children's everyday life | | 1 | 5 | 5 | 4 | 3.8 | 4 | | |
| When I was on a tour of the institution, I got a really good impression of the house, staff and manager. Many employees had been there for many years, which made me assume that it was a good workplace with satisfied employees | | | 2 | 9 | 4 | 4.1 | 4 | | |

| | | | | | | | | | |
|---|---|---|---|---|---|---|---|---|---|
| We noticed the adults' commitment and approach to the children. How are the children talked to and understood if, for example, they are sad. | | | 2 | 7 | 6 | 4.3 | 4 | | |
| The staff had great sensitivity to the children, and a good understanding of each child | | 1 | 3 | 4 | 7 | 4.1 | 4 | | |
| That there was good communication between staff and parents | 1 | | 4 | 7 | 3 | 3.7 | 4 | | |
| That the kindergarten teachers seemed happy and spoke warmly about the kindergarten, the colleagues and the management | | | 2 | 5 | 8 | 4.4 | 5 | | |
| There are many young kindergarten teachers employed, and I have the impression that everyone has an influence on everyday life, even the kindergarten assistants, who often are employed for a long time. | 3 | 3 | 4 | 3 | 2 | 2.9 | 3 | | |
| **PHYSICAL FRAMEWORK** | | | | | | | | | |
| Rhythm once a week and tumble room | 2 | 3 | 7 | 3 | | 2.7 | 3 | | |
| Cozy and manageable playground with fireplace | | 6 | 4 | 5 | | 2.9 | 3 | | |
| Small cozy institution with few groups of children. | 6 | 3 | | 6 | | 2.4 | 2 | | |
| The indoor facilities | | 1 | 5 | 9 | | 3.5 | 4 | | |
| That there were good outdoor areas | | | 6 | 7 | 2 | 3.7 | 4 | | |
| It was positive that the kindergarten had access to a house in a forest, where they some times whent, since we were not able or did not want to choose an outdoor kindergarten. | 5 | 6 | 2 | 2 | | 2.1 | 2 | | |
| Whether the decor (both outside and inside) was appropriate so that both nursery and kindergarten children are taken into account (in an integrated institution) | | 3 | 5 | 5 | 2 | 3.4 | 3 | | |
| That there were proper outdoor areas attached to the institution so that the children could run in and out to the playground | | 2 | 3 | 8 | 2 | 3.7 | 4 | | |
| Important with a focus on activity rooms in the institution such as workshops, sensory rooms, tumble rooms etc. | | 4 | 6 | 3 | 2 | 3.2 | 3 | 3.0 | 3 |
| Outdoor area such as a playground that invites to play with "hiding places" as well as green areas | | | 3 | 11 | 1 | 3.9 | 4 | | |
| That the physical setting provided opportunities for different and undisturbed play in the design of different play nooks | 1 | 2 | 6 | 4 | 2 | 3.3 | 3 | | |
| That there was a workshop and a hall, for the development of creativity, gymnastics and physical activity | 3 | 2 | 5 | 3 | 2 | 2.9 | 3 | | |
| That the physical spaces were homely, cozy and vibrant | | 2 | 4 | 8 | 1 | 3.5 | 4 | | |
| That the rooms in the kindergarten seemed personal with many pictures of the children, framed drawings, creative projects | 1 | 4 | 3 | 6 | 1 | 3.1 | 3 | | |
| That the kindergarten had an outdoor kitchen so they could cook for the kids on the playground | 8 | 6 | 1 | | | 1.5 | 1 | | |

| | | | | | | | | | |
|---|---|---|---|---|---|---|---|---|---|
| That the kindergarten was in close proximity to the city oasis, playgrounds, parks, etc. | 6 | 5 | 2 | 1 | 1 | 2.1 | 2 | | |
| The kindergarten had a welcoming setting - for adults and children. It was newly renovated inside and out with sustainable and accommodating materials and creative solutions for children! | 2 | 3 | 6 | 3 | 1 | 2.9 | 3 | | |
| The institution is located in a nice green area, even though it is in the middle of Nørrebro | 3 | 1 | 7 | 2 | 2 | 2.9 | 3 | | |
| The institution is located in a beautiful old but newly renovated building with a nice big playground | 5 | 4 | 3 | 3 | | 2.3 | 2 | | |
| **STRUCTURE / PEDAGOGICAL VALUES / FOOD POLICY** | | | | | | | | | |
| Non-rigid sugar policies etc. | 3 | 4 | 6 | 2 | | 2.5 | 3 | **3.2** | **3** |
| It seemed that there was a plan for the day and there was a clear division of roles between the kindergarten teachers. | 2 | 2 | 3 | 7 | 1 | 3.2 | 4 | | |
| The distribution of roles works really well, that e.g. is someone who immerses himself with the kids and someone who arranges the food and diaper changes | 5 | 2 | 4 | 3 | 1 | 2.5 | 3 | | |
| The distribution of roles works really well, e.g. someone immerses himself with the kids and someone arranges the food and diaper changes | | 2 | 7 | 4 | 2 | 3.4 | 3 | | |
| It is reassuring if you can read on the institution's website what considerations they have about the contain of the day for the children. | 3 | 2 | 6 | 4 | | 2.7 | 3 | | |
| Organic food, made in the kitchen of the institution | 3 | 1 | 4 | 6 | 1 | 3.1 | 3 | | |
| That they provided plenty of space for the children's play | 1 | | 4 | 9 | 1 | 3.6 | 4 | | |
| That it was a kindergarten with room for children with special needs and extensive experience of working with inclusion and diversity. | 2 | 7 | 3 | 3 | | 2.5 | 2 | | |
| That there was a special focus (on creativity and art) was positive | 2 | 3 | 4 | 5 | 1 | 3.0 | 3 | | |
| There was a chef attached who made delicious hot and organic food for the entire kindergarten. | 3 | 2 | 2 | 7 | 1 | 3.1 | 4 | | |
| Food arrangement in the kindergarten is a plus. | 1 | 2 | 3 | 6 | 3 | 3.5 | 4 | | |
| Good traditions, and a clear culture | | 3 | 5 | 4 | 3 | 3.5 | 3 | | |
| That the kindergarten / staff had clear pedagogical, social values, which were followed in practice | | 1 | 3 | 7 | 4 | 3.9 | 4 | | |
| That the staff had a lot of focus on the children's social relationships | | 1 | 2 | 6 | 6 | 4.1 | 4 | | |
| That the kindergarten emphasized going out for walks, and among other things used the local gym hall regularly weekly | | 2 | 8 | 4 | 1 | 3.3 | 3 | | |
| That there was a focus on presence and security, eg by prioritizing activities in smaller, age-appropriate groups | | 2 | 1 | 8 | 4 | 3.9 | 4 | | |
| That the kindergarten emphasized creative activities | 1 | 2 | 7 | 5 | | 3.1 | 3 | | |

| | | | | | | | | | |
|---|---|---|---|---|---|---|---|---|---|
| **SHIFTS** | | | | | | | | | |
| We chose an integrated institution which we liked, with the hope that he could continue in the kindergarten in the same institution | 1 | 2 | 3 | 4 | 5 | 3.7 | 4 | | |
| That many from the same kindergarten will go on to the same primary school | 2 | 4 | 5 | 2 | 2 | 2.9 | 3 | | |
| I chose a nursery with integrated kindergarten. It is difficult to get into the desired kindergarten if you have not gone to the nursery that belongs to the integrated kindergarten | 5 | 1 | 4 | 3 | 2 | 2.7 | 3 | 3.2 | |
| That there was cooperation with the local primary school in connection with the transition between kindergarten and school | 3 | 3 | 3 | 4 | 2 | 2.9 | 3 | | 4 |
| That the institution was integrated so that the change from nursery to kindergarten could take place easily | 3 | 1 | 2 | 3 | 6 | 3.5 | 4 | | |
| That the kindergarten belongs to the same district as the school we wanted | 1 | 3 | 2 | 6 | 3 | 3.5 | 4 | | |
| **LOGISTICS** | | | | | | | | | |
| We thought it was too inflexible with fixed drop-off and pick-up time by the bus when going to an outdoor kindergarten, so the new kindergarten is not an outdoor kindergarten. | 3 | | 4 | 2 | 6 | 3.5 | 4 | | |
| We chose an institution that was close by so that our son would not have to be transported through a lot of traffic back and forth | | 2 | 3 | 6 | 4 | 3.8 | 4 | | |
| We opt out of outdoor kindergartens to have more flexibility in terms of dropping off and picking up at different times and to avoid long drives. | 4 | 1 | 1 | 5 | 4 | 3.3 | 4 | | |
| It was too much trouble with an outdoor kindergarten which is far away, and that you could only pick up by car if the child for some reason had to go home earlier. | 3 | 2 | | 5 | 5 | 3.5 | 4 | 3.5 | 4 |
| That it was close to our home, so that the logistics was easy in everyday life | | 1 | 2 | 5 | 7 | 4.2 | 4 | | |
| That it was not an outdoor kindergarten, as I would like to be able to pick up early a few times a week | 3 | 4 | 1 | 2 | 5 | 3.1 | 3 | | |
| That the institution was located near our residence | | | 2 | 5 | 8 | 4.4 | 5 | | |
| That it was an integrated institution (nursery and kindergarten integrated) so that the little brother would also be able to start in the nursery in the same institution. | 6 | 2 | 2 | 4 | 1 | 2.5 | 2 | | |
| **REPUTATION** | | | | | | | | | |
| I had read that it was an institution with a good reputation and then one day I asked a mother, outside the institution, how she experienced the place - and she spoke warmly about the institution | 1 | 3 | 6 | 4 | 1 | 3.1 | 3 | | 3 |
| That I had heard well about the institution from others | | 1 | 7 | 5 | 2 | 3.5 | 3 | | |
| Gut feeling - we chose an institution despite its reputation | 1 | | 7 | 5 | 2 | 3.5 | 3 | 2.9 | |

| | | | | | | | | | |
|---|---|---|---|---|---|---|---|---|---|
| That I have recommendations from friends and acquaintances who share the same view on childcare as I. | | 4 | 6 | 3 | 2 | 3.2 | 3 | | |
| I read thoroughly on the institution's website incl. supervision reports etc. | 4 | 6 | 5 | | | 2.1 | 2 | | |
| The most important are recommendations from other parents about the institution | 1 | 2 | 7 | 3 | 2 | 3.2 | 3 | | |
| We looked at the parental satisfaction survey | 7 | 3 | 3 | 1 | 1 | 2.1 | 2 | | |
| We were recommended the kindergarten by a kindergarten teacher who works in the municipality with supervision of kindergartens in Copenhagen | 7 | 2 | 3 | 1 | 2 | 2.3 | 2 | | |
| It is a well-run institution with a really good reputation (and long waiting list) | | 4 | 5 | 3 | 3 | 3.3 | 3 | | |
| **CHILD-TO-STAFF-RATIO** | | | | | | | | | |
| Small kindergarten with good rating of child per employee due to extra personnel for children with special needs. The rating gave great freedom and flexibility for spontaneous trips. | 6 | 1 | 5 | 3 | | 3.1 | 3 | 2.9 | 4 |
| The size mattered - that it was not a very big institution | 5 | | 2 | 7 | 1 | 3.5 | 3 | | |
| The rating of personnel per child is important, we chose a place with social rating and thus more adults per. child | 2 | 3 | 4 | 5 | 1 | 3.5 | 3 | | |
| the number of children compared to the number of adults to be with the children. | 2 | | 1 | 11 | 1 | 3.2 | 3 | | |
| That the institution is not too big with too many children | 4 | | 3 | 7 | 1 | 2.1 | 2 | | |
| That the children in the nursery were relatively old (+2) due to a long waiting list and that it seemed to give a good harmony in the group, because the children typically had language and were a little less demanding. Thereby, there was also time for creative projects | 7 | 4 | 3 | | 1 | 3.2 | 3 | | |
| *The means are based on the rating of importance of each statement on a 5-point scale, from 1 (‘not important’ for choosing kindergarten) to 5 (‘very important’). | | | | | | | | | |
