# Supplementary material for: Types of kindergarten and their relationship with parental and children’s socio-demographic characteristics in Denmark
Source: PLoS One. 2023 Jul 20;18(7):e0288846. doi: 10.1371/journal.pone.0288846 (PMC10358886; doi:10.1371/journal.pone.0288846)
Supplement: S2 Table — All statements were generated, ranked and divided into concepts by the parents from children attending outdoor kindergartens. (PDF) [file pone.0288846.s002.pdf]

Supplementary table 2. Conceptual model. All statements were generated, ranked and divided into concepts by the parents from children attending outdoor kindergartens.

Thinking as broadly as you can, please list your thoughts regarding which considerations you had when choosing a kindergarten for your child?	The Frequency of ranking*					Statement		Cluster	
**Statements**	**1**	**2**	**3**	**4**	**5**	**Mean**	**Median**	**Mean**	**Median**
**STRUCTURE AND ORGANIZATION**									
The structure and predictability of an everyday life that takes place away from parents / pick up and bring.		4	3	5	1	3.2	3	2.9	3
Flat management structure (impression: healthy environment)	1	2	7	2	1	3.0	3		
a kindergarten that has a child-safe area, and does not have areas that are dangerous for children and adults	1	3	6	2	1	2.9	3		
A relaxed approach to rules, and a not too structured set-up	2	3	4	4		2.8	3		
Parental involvement		3	8	2		2.9	3		
By bus to the outdoor kindergarten, and home again. Thus, no disturbances in the form of parents picking up and bringing.	4	2	2	4	1	2.7	3		
**LEARNING, FREEDOM AND PEDAGOGICAL IDEOLOGY**									
On the children's terms.		2	8	3		3.1	3	3.4	4
Playing, smiling, and attentive adults.				5	8	4.6	5		
Action behind the words in relation to the child's learning.		2	4	6	1	3.5	4		
Divided zones for play activities	3	8	2			1.9	2		
Focus on posters and other information relevant for the children.	4	4	5			2.1	2		
The experience that the adults respect the children.			1	7	5	4.3	4		
The experience that the children get lots of co-decision.	1	1	9	2		2.9	3		
Healthy approach on food.			4	8	1	3.8	4		
Creativity - offers a creative space for development.		2	4	6	1	3.5	4		
The children's co-decision as to whether they want to be inside or outside	2		8	3		2.9	3		
Whether the children may be included or not	1	1	5	6		3.2	3		
Good communication with the kindergarten teachers / management			2	6	5	4.2	4		
Kindergarten teachers with a focus on well-being				5	8	4.6	5		
Kindergarten teachers and their approach to children			1	3	9	4.6	5		
A good dietary plan.	1		5	5	2	3.5	4		
the most important thing for me was whether I could see my child and our family in the value and activities of the kindergarten	1	1	4	6	1	3.4	4		
What the focused is in the developmental plans (for the children)		2	7	3	1	3.2	3		
Mixed composition of children		4	8	1		2.8	3		
Which activities are in focus?	1	1	7	3	1	3.2	3		
**PHYSICAL SETTING WITH A FOCUS ON OUTDOOR LIFE AND FREEDOM OF MOVEMENT**									
The surroundings in the outdoor kindergarten: That there is both a large spacious and private outdoor area where the children can unfold and feel at home			1	9	3	4.2	4		

The surroundings in the outdoor kindergarten: That there is a house with good indoor conditions, so that children and staff can switch between inside and outside.			2	**7**	**4**	4.2	4		
Nature; see other than playgrounds in the area		1	1	6	**5**	4.2	4		
Nature (motor skills, play, freedom, mental and physical strength)			1	**8**	**4**	4.2	4		
Important and natural outdoor initiatives.			3	8	2	3.9	4		
A kindergarten where it is nice to be and where the decor and surroundings have been thought through.		2	4	7		3.4	4		
Annual cycle with repetitions and a focus on security, predictability and development.	2	3	2	6		2.9	3		
Less chaotic everyday life with the opportunity to be outside and with an opportunity to voluntarily retire if necessary.			1	**9**	**3**	4.2	4		
Opportunity to be more outside than inside			2	7	4	4.2	4		
The bus ride to and from the kindergarten is long, which is not optimal, but OK in relation to how good a place they are going to.	3	2	4	2	2	2.8	3		
Outdoor kindergarten. We live in an apartment in the city. The kindergarten can give something other than we can give		1	8	4		4.2	4		
Lots of space for children to hide and play alone	1	2	2	7	1	3.4	4		
Big outdoor area		1		10	2	4.0	4	3.9	4
The children are allowed to roll in mud, so they are dirty right up to their ears. Be allowed to be an active child.			2	7	4	4.2	4		
Nature and fresh air away from the city			2	5	6	4.3	4		
Activity, space to run, climb trees, dig, jump, etc.			1	7	5	4.3	4		
Out of town and see the reality of nature.		1	2	6	4	4.0	4		
Feel, smell and see the change of seasons		1	3	3	6	4.1	4		
Healthy for body and mind to be outdoors			2	6	5	4.2	4		
More exercise and less illness			3	8	2	3.9	4		
More space and thus fewer conflicts		1	2	7	3	3.9	4		
More nature in the everyday life			3	5	5	4.2	4		
Have read that children in outdoor kindergartens generally have better motor skills and are generally less ill (perhaps a little secondary) in relation to the idea of fresh air and outdoor life as an overall ideal.		1	1	9	2	3.9	4		
Healthy not to be under supervision all the time, but to be able to stay outdoors on a large area, where you can also go to and from play and spend time with other children - and adults.	1		6	4	2	3.5	3		
To give our son a proper shot of nature by choosing the outdoor kindergarten, now that we have chosen to live in the city. Nature understood as being able to rummage in soil, climb trees, look at insects and animals (chickens - and now and then the fox's ravages), plants, outdoors		1	1	4	7	4.3	5		
Being in the outdoor kindergarten on a large area gives the children the opportunity to be on their own and invent their own activities without constant adult supervision.		1	4	4	4	3.8	4		
Noise fits better outdoors. It is natural for children to shout, make noise, run, etc.	1	1	4	4	3	3.5	4		
**EXPERIENCES**									
Proper evaluation in the annual inspection report from the municipality	1	5	4	2	1	2.8	3		

Recommendations from others.		3	3	5	2	3.5	4		
Not too big a day care institution	3	2	1	6	1	3.0	4		
Wanted a small kindergarten in relation to the number of children.	5	1	2	4	1	2.6	3		
Other parents' recommendations	1	3	2	6	1	3.2	4		
Recommendations from friends and acquaintances whose children have attended the institution themselves (recently) and I like both the environment and the educational work.	1	2	3	5	2	3.4	4		
I attended an outdoor kindergarten myself as a child.	9	2	1	1		1.5	1	2.8	3
The institutions (not outdoor kindergartens) we were offered were not attractive	5	2	4	2		2.2	2		
That there would probably be many other children from the local area who go to the same outdoor kindergarten, so that friendships could be made - perhaps all the way to and during the school transition.	2	3	4	4		2.8	3		
**EVERYDAY LIFE**									
Had a plan of picking up directly at the outdoor kindergarten a little more often, now that it's only 20 minutes away.	6	3	2	2		2.0	2		
Should be close to our accommodation		2	6	3	2	3.4	3		
Distance to residence when all the important things are in place		3	6	3	1	3.2	3		
location of the institution in relation to home and work		2	4	5	2	3.5	4		
available space at the desired start-up time	1	2	5	3	2	3.2	3		
Opening hours.	1	6	3	2	1	2.7	2		
Close to home.		2	5	3	3	3.5	3		
Practical - what is best in relation to making our everyday life cohesive.		1	5	4	3	3.7	4	3.2	3
Location (relative to home / work)	1	1	4	3	4	3.6	4		
That the outdoor kindergarten is not more than 20 minutes away from the but meeting point - we do not want our son to sit too long in a bus every day (would not want a long transportation time every day our self).	3	3	2	2	3	2.9	3		
Short distance from home to the bus meeting point, so that everyday life can be as easy in terms of logistics as possible.		2	5	3	3	3.5	3		
**PERSONNEL**									
Got a good impression of the kindergarten teachers during a visit to the institution.			1	6	6	4.4	4		
I experience, that the kindergarten teachers have a healthy and natural relationship with the children.			1	4	8	4.5	5		
The kindergarten teachers, humor, attention, accessibility and warmth.				6	7	4.5	5		
Leader with long seniority		6	6	1		2.6	3		
Stable staff with long seniority			3	8	2	3.9	4	3.8	4
Honest and approachable kindergarten teachers.			2	7	4	4.2	4		
Present kindergarten teachers.				5	8	4.6	5		
Whether the communication with the institution was good and clear - it was a bit difficult to determine due to covid-19.		1	5	6	1	3.5	4		
Can I relate to the manager of the place?	1	4	6		2	2.8	3		
What is the employee turnover (is it a place where the employees like to be, or do they slip away quickly)		1	5	4	3	3.7	4		

What is the absence rate (among kindergarten teachers)		5	3	4	1	3.1	3		
Good impression of the staff group, it seemed cooperative, dynamic and really good at helping each other and receiving children and parents by the bus.	1			5	7	4.3	5		

*The means are based on the rating of importance of each statement on a 5-point scale, from 1 (‘not important’ for choosing kindergarten) to 5 (‘very important’).
