# Supplementary material for: Types of kindergarten and their relationship with parental and children’s socio-demographic characteristics in Denmark
Source: PLoS One. 2023 Jul 20;18(7):e0288846. doi: 10.1371/journal.pone.0288846 (PMC10358886; doi:10.1371/journal.pone.0288846)

Supplementary Figure 1. The optimal cluster soution for statements generated among parents with children attending Outdoor kindergartens.

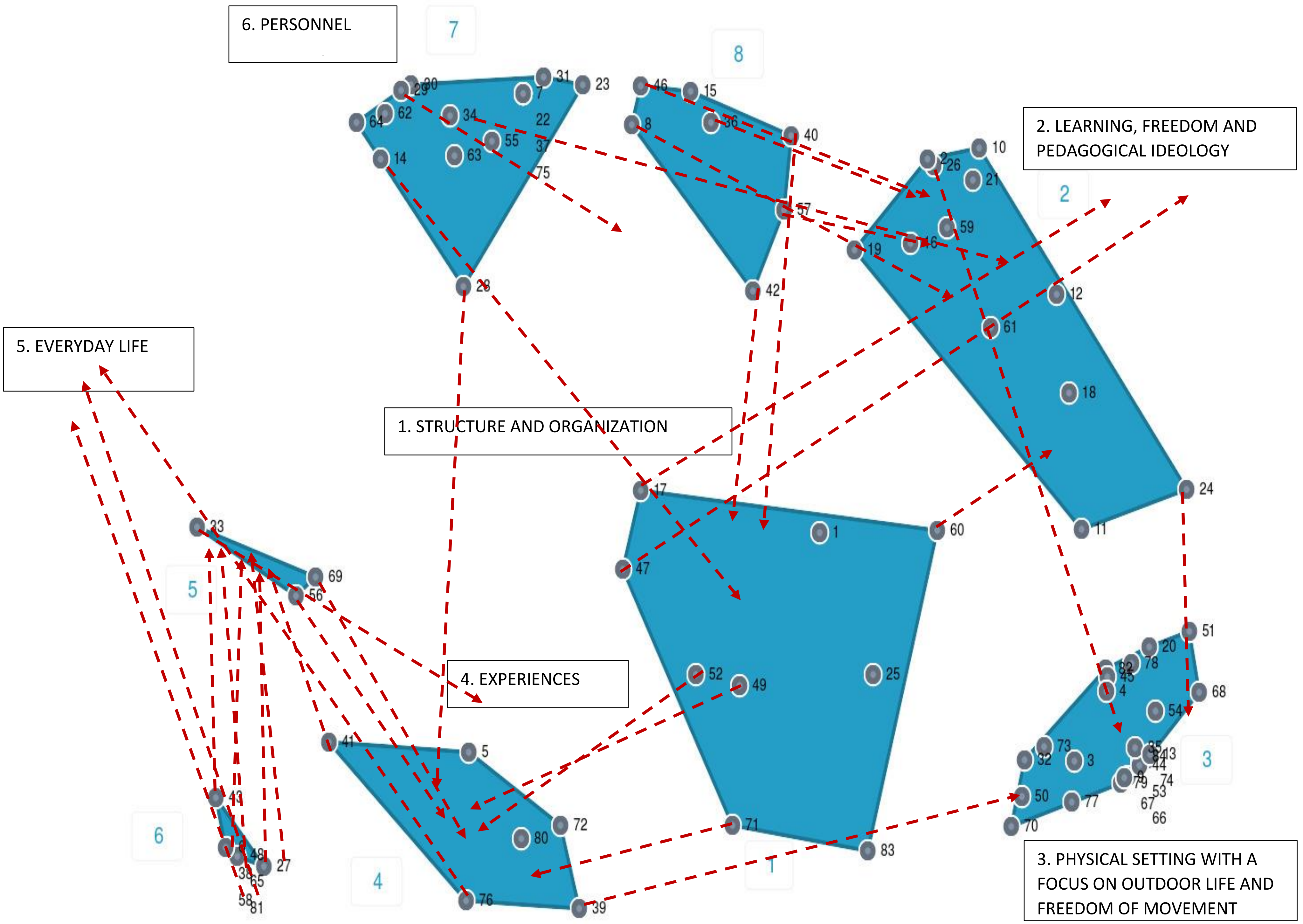

Supplement: S1 Fig — (PDF) [file pone.0288846.s003.pdf]
